# Subangular Deep Fascia Fixation for Submental U-Shaped Cogged Thread Lifting

**DOI:** 10.1016/j.jpra.2026.04.028

**Published:** 2026-05-08

**Authors:** Gi-Woong Hong, Kyu-Ho Yi

**Affiliations:** a Samskin Plastic Surgery Clinic, Seoul, South Korea; b You and I Clinic, Seoul, Korea

**Keywords:** Thread lifting, Cogged thread, Double chin, Antegonial notch, Great auricular nerve, Subangular deep fascia

## Abstract

**Background:**

Submental “double-chin” deformity is multifactorial and may reflect the volume and boundaries of preplatysmal fat, variability in platysmal decussation, and aging-related changes at the cervicomental angle. U-shaped cogged thread lifting is increasingly used as a minimally invasive option for submental contouring, yet the mandibular angle/antegonial notch region contains facial vessels and nerve branches that can be injured when the cannula or thread deviates into a deeper plane.

**Objectives:**

To describe cadaveric anatomical observations relevant to submental U-shaped cogged thread lifting and to propose a fixation concept using a regionally condensed fibrous layer beneath the platysma near the mandibular angle (herein referred to as the “subangular deep fascia”), representing a functional fascial interface rather than a formally defined anatomical layer, to reduce off-target traversal and potential injury.

**Methods:**

Cadaveric thread simulations were performed to reproduce common U-shaped submental thread trajectories. Layer-by-layer dissections were used to identify the relationship of the simulated thread path to the platysma, facial artery/vein at the antegonial notch region, parotid tail, sternocleidomastoid muscle, and superficial sensory nerves. Representative photographs were compiled as figures.

**Results:**

When the trajectory was directed toward a posterior mastoid fixation, the thread pathway tended to approach deeper tissue planes near the mandibular angle, placing it in close proximity to vertically ascending facial vessels; a representative specimen demonstrated direct arterial penetration. A relatively thick and resistant fibrous layer was consistently appreciable beneath the platysma near the mandibular angle, which may represent a regional fascial condensation rather than a distinct named anatomical structure, and provided a plausible alternative fixation point. Conceptually, a subangular deep fascia fixation corridor allows the thread to remain predominantly preplatysmal while limiting posterior traversal through the parotid–sternocleidomastoid region.

**Conclusions:**

The mandibular angle/antegonial notch region represents a critical transition zone in submental thread lifting. Anchoring within a dense subangular deep fascial layer beneath the platysma may improve procedural safety by reducing the need for deep posterior passage and by supporting a stable lifting vector. Further quantitative cadaveric mapping and prospective clinical studies are needed.

## Introduction

Submental “double-chin” deformity is multifactorial and reflects not only preplatysmal fat volume but also septal boundaries, platysmal morphology/decussation, and ageing-related changes of the cervicomental angle. Absorbable U-shaped cogged thread lifting is increasingly used as a minimally invasive option for submental contouring; however, reported complications include contour irregularity, dimpling, paresthesia, hematoma, infection, and thread extrusion. In clinical practice, a key technical challenge is maintaining a consistent preplatysmal plane while traversing the mandibular angle/antegonial notch region, where resistance increases and the cannula or thread may inadvertently enter deeper planes. This area is clinically important because the facial vessels may course close to mandibular landmarks and deeper passage can encroach on the marginal mandibular nerve risk zone. In addition, posterior suspension concepts that require extended travel toward a mastoid anchor may traverse the parotid tail and sternocleidomastoid (SCM) corridor, where the great auricular nerve is relatively superficial.

**Figure 1:**
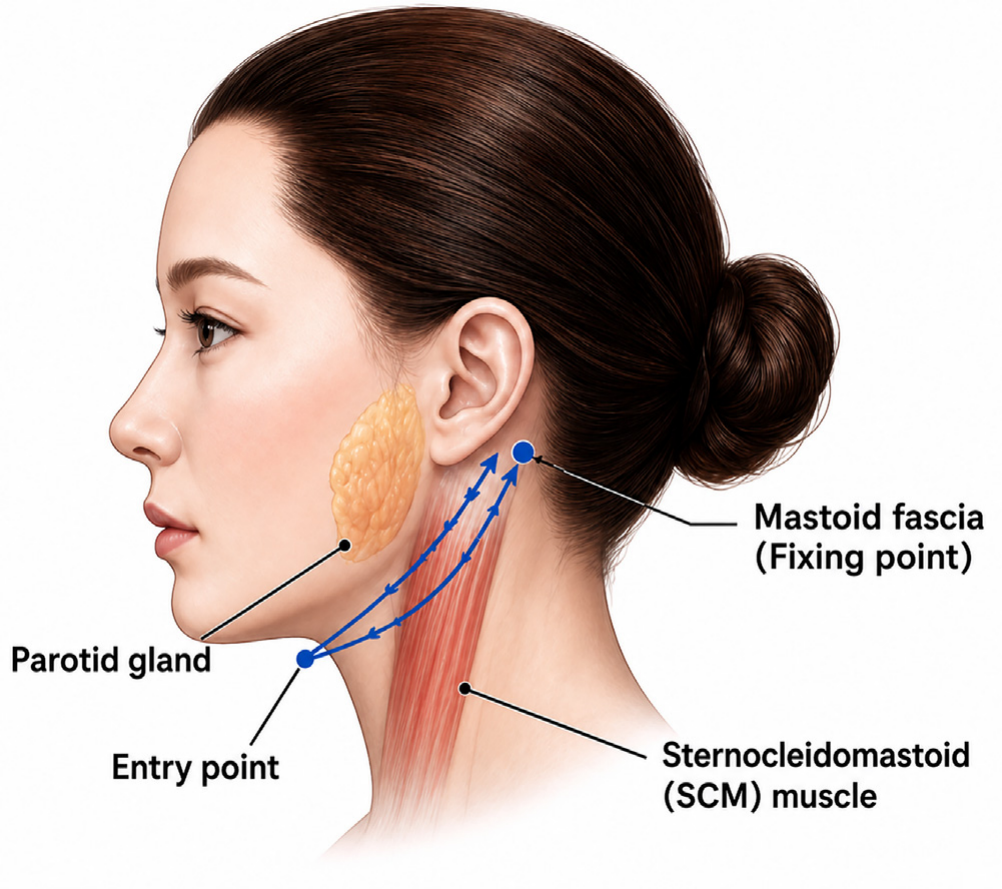
Conventional concept of a long U-shaped cogged thread anchored to mastoid fascia for submental lifting. The posterior passage can traverse the parotid tail and the sternocleidomastoid region, where superficial sensory nerves and the external jugular vein may be encountered. Sihler Thread has been used.

The present technical note summarizes cadaveric observations from simulated submental U-shaped cogged thread trajectories and proposes a local fixation concept using a dense fibrous layer beneath the platysma near the mandibular angle (“subangular deep fascia”) to reduce off-target posterior traversal and potential injury (Figure 1).

## Materials and methods

**Figure 2:**
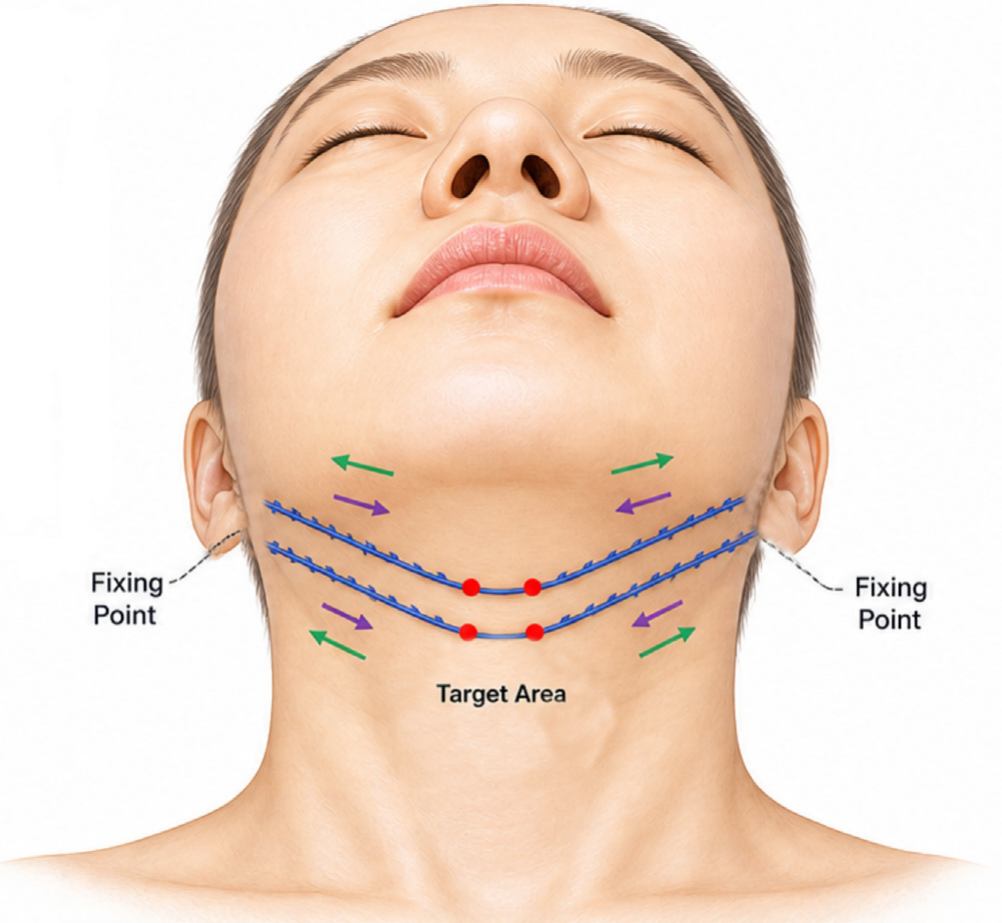
Example design of a submental U-shaped long cogged thread intended to elevate preplatysmal soft tissue and improve the cervicomental angle. Sihler Thread has been used.

This descriptive cadaveric anatomical technical note used donated head-and-neck specimens in accordance with local institutional policies governing anatomical donation and research use; no identifying information was retained. Using a blunt cannula, we simulated placement of a long U-shaped cogged thread from an anterior submental entry site. The cannula was advanced laterally within the intended preplatysmal soft tissue to cross the mandibular angle/antegonial notch region. Two fixation strategies were examine:d.^1^ posterior mastoid-directed fixation, consistent with traditional suspension-type descriptions, and.^2^ local fixation within a dense fibrous layer beneath the platysma near the mandibular angle (subangular deep fascia) (Figure 2).

After simulation, stepwise dissections were performed to expose the skin/subcutaneous layer, platysma, and deeper planes. The relationship of the simulated track to key structures (facial artery/vein near the antegonial notch, parotid tail, SCM region, and superficial sensory nerve territory including the great auricular nerve) was documented photographically. Representative images were compiled, with key illustrations presented in the main manuscript and additional images provided as supplementary material.

## Results

Across specimens, the mandibular angle/antegonial notch region consistently acted as a critical transition zone where tactile resistance increased; when a steep posterior vector was pursued, the simulated pathway could unintentionally descend into deeper planes. In mastoid-directed simulations, the track tended to course toward the parotid tail–SCM corridor, a region where superficial sensory nerves are vulnerable and where major veins may be encountered. Within the antegonial notch region, the simulated path frequently lay immediately adjacent to vertically ascending facial vessels; in a representative demonstration, direct arterial penetration was observed (see Supplementary Figures), illustrating a plausible mechanism for bleeding during thread placement when depth control is lost.

A distinct dense fibrous tissue layer was consistently appreciable beneath the platysma near the mandibular angle. This layer offered higher resistance to blunt dissection than surrounding tissue and appeared continuous with deeper cervical fascial structures. When the fixation concept was shifted to this subangular deep fascial layer, the simulated trajectory could be maintained predominantly within the preplatysmal plane across the mandibular angle/antegonial notch, with anchoring achieved without extended posterior traversal through the parotid–SCM corridor.

## Discussion

These cadaveric simulations reinforce that submental U-shaped thread lifting is a plane-dependent procedure, and the mandibular angle/antegonial notch represents a high-stakes transition where loss of depth control can place the cannula/thread close to facial vessels and within the marginal mandibular nerve risk zone. The observed vessel proximity and penetration (see Supplementary Figures) provide a concrete anatomic explanation for unexpected bleeding or expanding hematoma when the track dives beneath the intended preplatysmal plane. From a procedural planning standpoint, the findings support two safeguards: (i) prioritize tactile and trajectory cues to remain in the intended preplatysmal corridor as resistance increases near the mandibular angle, and (ii) select a fixation strategy that minimizes unnecessary deep posterior travel when feasible.

Posterior suspension concepts that require prolonged travel toward a mastoid anchor may increase exposure to the parotid tail–SCM corridor, where the great auricular nerve is relatively superficial. Although thread lifting is less invasive than open procedures, the same surface anatomy may predispose to nerve irritation or injury if the cannula traverses this corridor at an unintended depth. Previous thread lifting techniques have commonly utilized posterior anchoring points such as the mastoid fascia or platysma-based suspension systems. While these approaches may provide strong lifting vectors, they often require extended posterior traversal through the parotid tail–sternocleidomastoid region, increasing the potential risk of encountering the great auricular nerve and major vascular structures. In contrast, the proposed subangular deep fascia fixation concept emphasizes a more localized anchoring strategy near the mandibular angle, potentially reducing the need for deep posterior passage while maintaining effective mechanical support. This conceptual shift may improve safety by limiting exposure to anatomically vulnerable zones.^3^, ^4^, ^5^

The proposed subangular deep fascia fixation concept offers an anatomic rationale to stabilize the lifting vector locally at the mandibular angle within a dense fibrous layer deep to the platysma, reducing the need for deep posterior passage. The term “subangular deep fascia” is introduced here as a conceptual and functional description rather than a formally established anatomical entity. Based on cadaveric observation, this region appears to correspond to a localized fascial condensation at the interface between the platysma-associated superficial fascia and deeper cervical fascial structures. While histological confirmation was not performed in this study, the consistent presence of a mechanically resistant fibrous layer supports its potential relevance as a practical anchoring substrate. Future anatomical and histological investigations are required to further characterize this structure and determine whether it represents a distinct anatomical layer or a region-specific variation of known fascial systems.

Limitations include its descriptive cadaveric design and lack of quantitative distance mapping. Further morphometric work is needed to define reproducible safe corridors for double-chin thread lifting.

## Ethics and disclosure

Cadaveric materials were used in accordance with institutional policies governing body donation and anatomical research. Formal ethical approval was waived as no identifiable human subject data were involved. This manuscript contains no identifiable patient information. The authors declare no conflicts of interest and received no external funding for this work.

## Funding

None.

## Financial disclosure

There is no financial disclosure to report.

## Ethical approval

Not required (cadaveric anatomical study conducted under institutional body donation policies).

## Informed consent

Not applicable.

## Author contributions

All authors have reviewed and approved the article for submission.

Conceptualization: Gi-Woong Hong

Methodology: Kyu-Ho Yi, Gi-Woong Hong

Investigation: Kyu-Ho Yi, Gi-Woong Hong

Data Curation: Gi-Woong Hong

Writing-Original Draft Preparation: Kyu-Ho Yi, Gi-Woong Hong

Writing-Review & Editing: Gi-Woong Hong

Visualization: Gi-Woong Hong

Supervision: Kyu-Ho Yi.

## Declaration of competing interest

The authors declare that they have no conflicts of interest to disclose.

## Appendix Supplementary materials

Supplementary Figure 1 Cadaveric and schematic illustration of key anatomical structures beneath the platysma around the mandibular angle/antegonial notch region, including the facial artery (FA), facial vein (FV), facial nerve (FN), masseter muscle (MM), great auricular nerve (GAN), and parotid gland. The angular tract is indicated to demonstrate the potential pathway of thread placement in relation to these structures. Image, image 1 dummy alt text

Supplementary Figure 2 Cadaveric simulation showing a thread/cannula trajectory placed in the deeper portion of the preplatysmal fat, immediately superficial to a vertically ascending facial vessel near the antegonial notch. The facial artery (FA) is indicated. Image, image 2 dummy alt text

Supplementary Figure 3 Cadaveric simulation after deeper dissection demonstrating the thread trajectory in close proximity to the ascending facial vessel in the antegonial notch region. The facial artery (FA) is indicated to emphasize anatomical risk. Image, image 3 dummy alt text

Supplementary Figure 4 Cadaveric finding demonstrating direct penetration of a vertically ascending facial artery at the antegonial notch region by the simulated thread trajectory. This illustrates a potential mechanism of vascular injury and hematoma formation during submental thread lifting when depth control is lost. Image, image 4 dummy alt text

Supplementary Figure 5 Cadaveric dissection demonstrating a dense fibrous layer beneath the platysma near the mandibular angle (proposed “subangular deep fascia”). This structure appears as a localized fascial condensation and may serve as a stable anchoring point for thread fixation. Image, image 5 dummy alt text

Supplementary Figure 6 Dissection of the retromandibular region illustrating two conceptual anchoring level:s.^1^ a superficial preplatysmal ‘hanging point’ and.^2^ a deeper fixation point within the proposed subangular deep fascia beneath the platysma, anterior to the sternocleidomastoid muscle. This comparison highlights the potential advantage of localized deep fascial fixation. Image, image 6 dummy alt text
