# Supplementary figures and images for: Subangular Deep Fascia Fixation for Submental U-Shaped Cogged Thread Lifting
Source: JPRAS Open. 2026 May 8;50:579–84. doi: 10.1016/j.jpra.2026.04.028 (PMC13279025; doi:10.1016/j.jpra.2026.04.028)

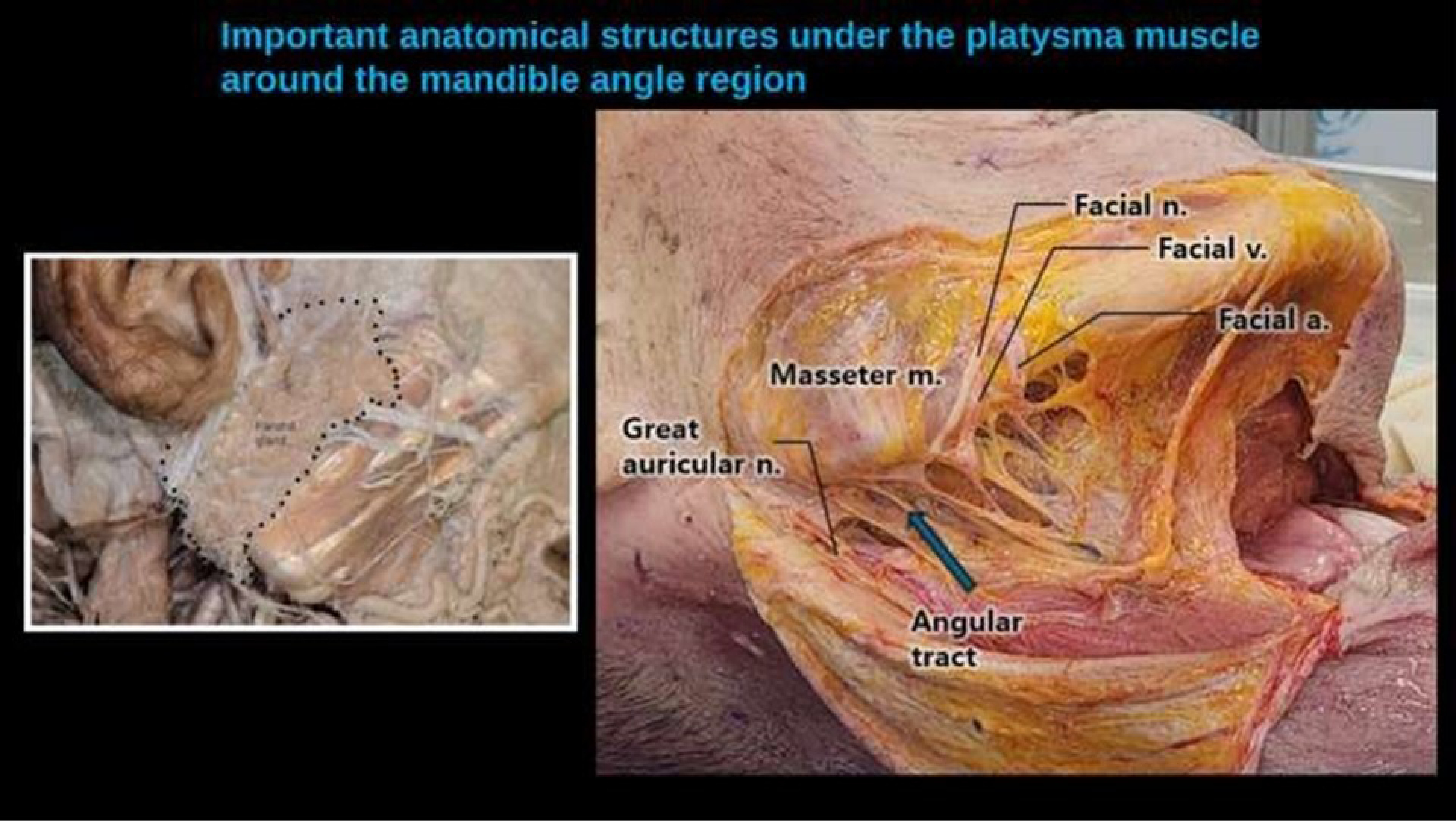

Supplement: Supplementary Figure 1 — Cadaveric and schematic illustration of key anatomical structures beneath the platysma around the mandibular angle/antegonial notch region, including the facial artery (FA), facial vein (FV), facial nerve (FN), masseter muscle (MM), great auricular nerve (GAN), and parotid gland. The angular tract is indicated to demonstrate the potential pathway of thread placement in relation to these structures. [file mmc1.jpg]

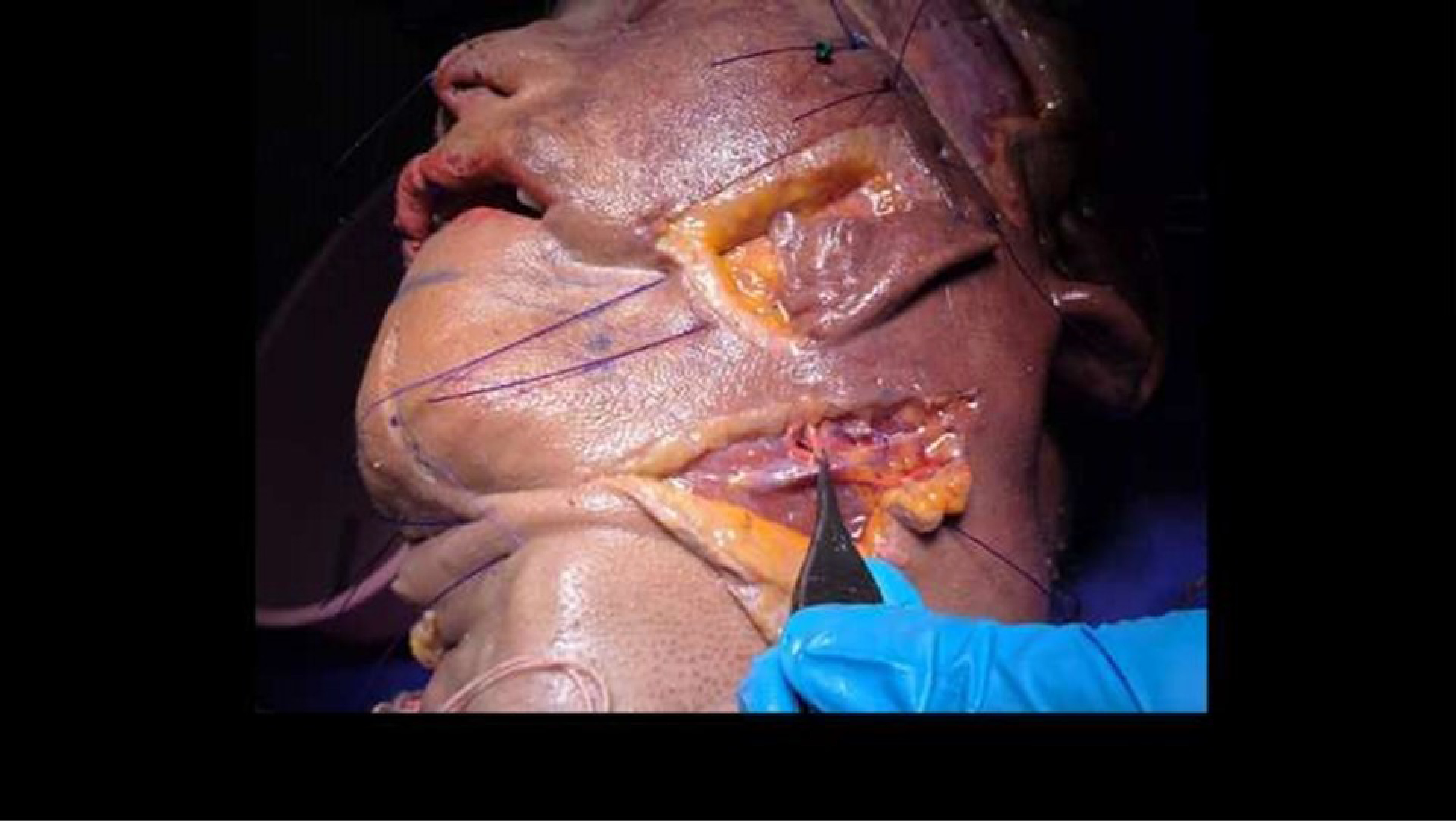

Supplement: Supplementary Figure 2 — Cadaveric simulation showing a thread/cannula trajectory placed in the deeper portion of the preplatysmal fat, immediately superficial to a vertically ascending facial vessel near the antegonial notch. The facial artery (FA) is indicated. [file mmc2.jpg]

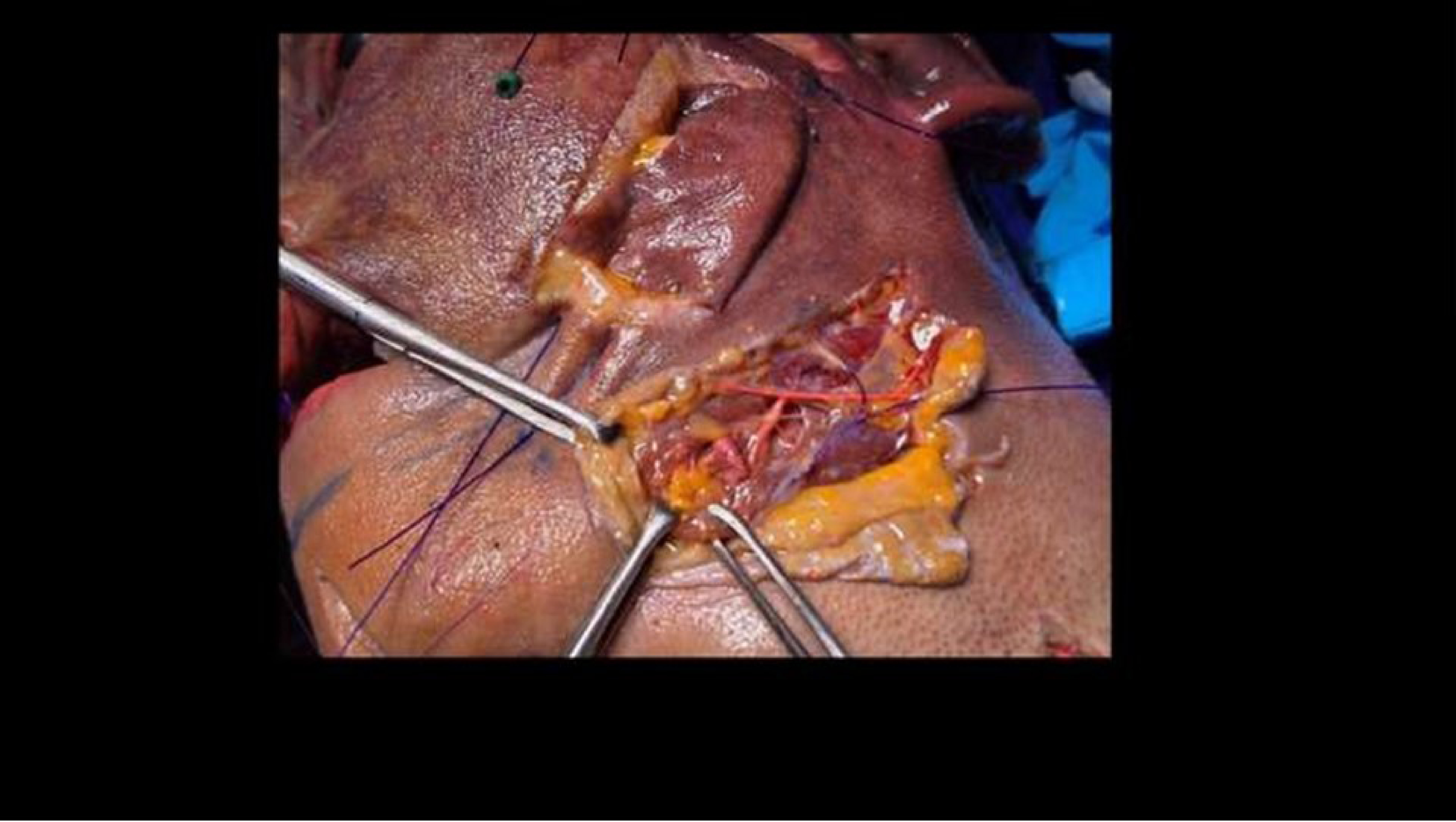

Supplement: Supplementary Figure 3 — Cadaveric simulation after deeper dissection demonstrating the thread trajectory in close proximity to the ascending facial vessel in the antegonial notch region. The facial artery (FA) is indicated to emphasize anatomical risk. [file mmc3.jpg]

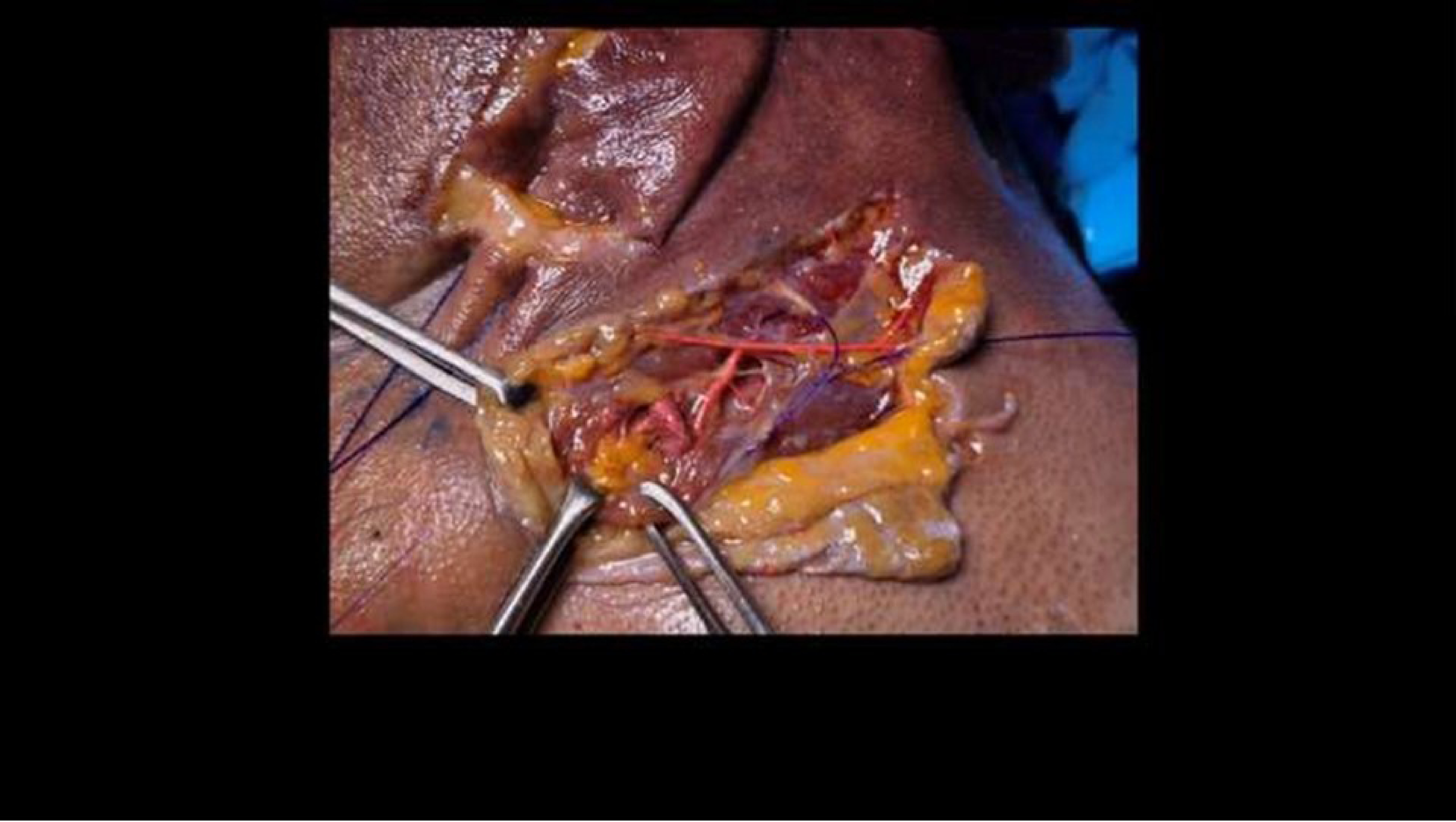

Supplement: Supplementary Figure 4 — Cadaveric finding demonstrating direct penetration of a vertically ascending facial artery at the antegonial notch region by the simulated thread trajectory. This illustrates a potential mechanism of vascular injury and hematoma formation during submental thread lifting when depth control is lost. [file mmc4.jpg]

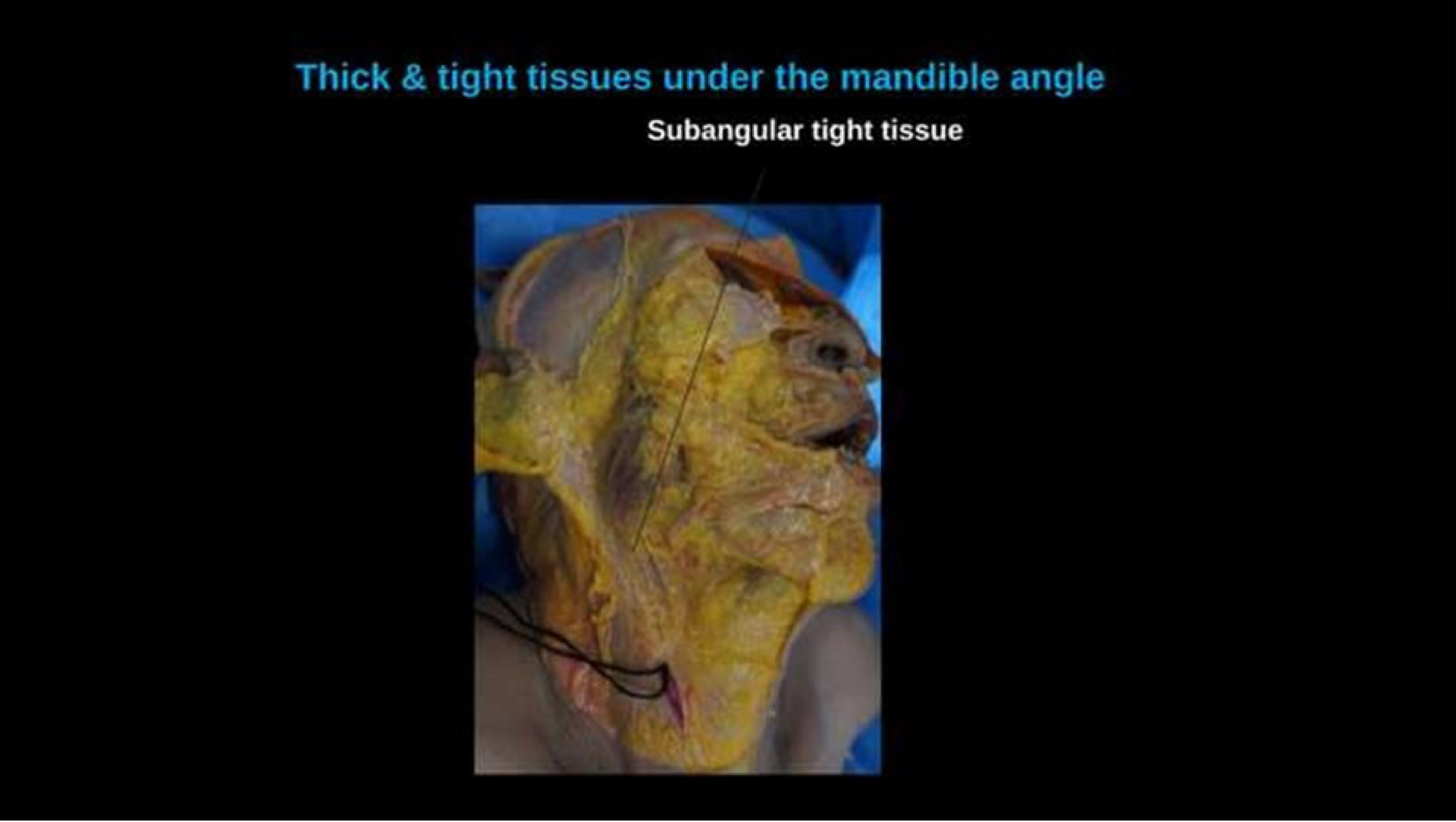

Supplement: Supplementary Figure 5 — Cadaveric dissection demonstrating a dense fibrous layer beneath the platysma near the mandibular angle (proposed “subangular deep fascia”). This structure appears as a localized fascial condensation and may serve as a stable anchoring point for thread fixation. [file mmc5.jpg]

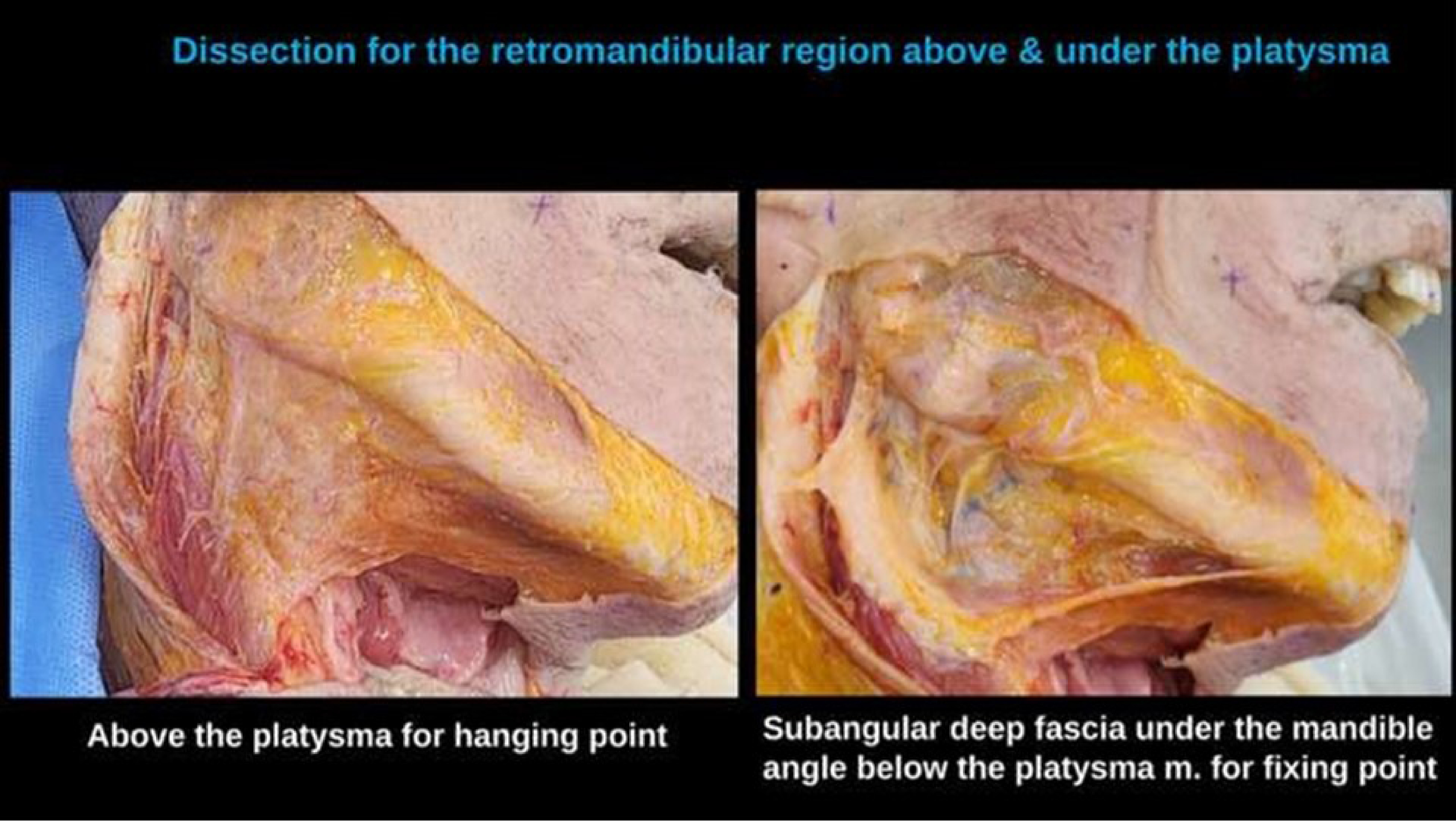

Supplement: Supplementary Figure 6 — Dissection of the retromandibular region illustrating two conceptual anchoring level:s.1 a superficial preplatysmal ‘hanging point’ and.2 a deeper fixation point within the proposed subangular deep fascia beneath the platysma, anterior to the sternocleidomastoid muscle. This comparison highlights the potential advantage of localized deep fascial fixation. [file mmc6.jpg]
